# De novo *TRPM3* missense variant associated with neurodevelopmental delay and manifestations of cerebral palsy

**DOI:** 10.1101/mcs.a006293

**Published:** 2023-12

**Authors:** Jagadish Chandrabose Sundaramurthi, Anita M. Bagley, Hannah Blau, Leigh Carmody, Amy Crandall, Daniel Danis, Michael A. Gargano, Anxhela Gjyshi Gustafson, Ellen M. Raney, Mallory Shingle, Jon R. Davids, Peter N. Robinson

**Affiliations:** 1 The Jackson Laboratory for Genomic Medicine, Farmington, Connecticut 06032, USA;; 2 Shriners Children's Northern California, Sacramento, California 95817, USA;; 3 Shriners Children's, Portland, Oregon 97239, USA;; 4 Shriners Children's Genomics Institute, Tampa, Florida 33612, USA;; 5 Department of Orthopaedic Surgery, University of California Davis Health, Sacramento, California 95817, USA;; 6 Institute for Systems Genomics, University of Connecticut, Farmington, Connecticut 06032, USA

**Keywords:** absent speech, bilateral convulsive seizures, bilateral talipes equinovarus, broad forehead, deeply set eye, intellectual disability, moderate, language impairment, moderate global developmental delay, thoracic scoliosis, torticollis

## Abstract

We identified a de novo heterozygous transient receptor potential cation channel subfamily M (melastatin) member 3 (*TRPM3*) missense variant, p.(Asn1126Asp), in a patient with developmental delay and manifestations of cerebral palsy (CP) using phenotype-driven prioritization analysis of whole-genome sequencing data with Exomiser. The variant is localized in the functionally important ion transport domain of the TRPM3 protein and predicted to impact the protein structure. Our report adds *TRPM3* to the list of Mendelian disease–associated genes that can be associated with CP and provides further evidence for the pathogenicity of the variant p.(Asn1126Asp).

## INTRODUCTION

Cerebral palsy (CP) is the most common motor disability with onset in childhood, affecting about 2 per 1000 children (Van Naarden Braun et al. 2016). CP is a group of nonprogressive disorders of movement and posture related to disturbances occurring in the developing fetal or infant brain. The motor disorders of CP are often accompanied by disturbances of sensation, perception, cognition, communication, and behavior, by epilepsy, and by secondary musculoskeletal problems (Rosenbaum et al. 2007). Growing evidence suggests manifestations of CP can be observed in individuals with Mendelian neurodevelopmental disorders (Friedman et al. 2022; Srivastava et al. 2022).

Transient receptor potential cation channel subfamily M (melastatin) member 3 (TRPM3) is a nonselective, structurally homotetrameric, calcium-permeable cation channel, and a member of the mammalian TRP family (Grimm et al. 2003; Mickle et al. 2015; Held and Tóth 2021). Functional TRP channels are either homotetramers of an individual channel member or heterotetramers formed by several members of the TRPC, TRPV, TRPM, and TRPP families (Grimm et al. 2003; Mickle et al. 2015; Held and Tóth 2021); TRPM3 forms functional heteromultimeric channels with TRPM1 protein (Lambert et al. 2011). The TRPM3 channel protein is known to be expressed in different tissues including human brain and kidney and plays an important role in calcium ion signaling, detection of noxious heat, and homeostasis (Grimm et al. 2003; Held et al. 2015). Pathogenic variants in *TRPM3* are associated with neurodevelopmental disorder with hypotonia, dysmorphic facies, and skeletal anomalies, with or without seizures (NEDFSS; OMIM:620224). NEDFSS is clinically characterized by neurodevelopmental delay, intellectual disability, hypotonia, motor delay and disability, speech impairments, and epilepsy (Dyment et al. 2019; de Sainte Agathe et al. 2020; Gauthier et al. 2021; Kang et al. 2021; Lines et al. 2022). However, manifestations of CP have not previously been reported in individuals with NEDFSS. In the present study, we report a de novo missense variant in the ion transport domain of TRPM3 in a child with neurodevelopmental delay and manifestations of CP.

## RESULTS

### Clinical Presentation and Family History

The subject of this paper is a 10-yr-old boy with multiple medical concerns including CP, gross motor delay, neurogenic dysphagia with aspiration, gastrostomy tube (G-tube) dependency, receptive expressive language delay, failure to thrive, visual impairment with myopic astigmatism, mild hyperopic amblyopia of the right eye, disconjugate gaze, optic nerve hypoplasia, knee and elbow flexion contractures, clasped thumbs, and camptodactyly of the fingers. For purposes of discussion, these concerns are divided into sections of systemic/neurologic and musculoskeletal, and a complete list of phenotypic features coding using the Human Phenotype Ontology (HPO) is provided in Table 1.

**Table 1.** Phenotypic features observed in the children who had variants in *TRPM3*

S#	HPO phenotypic terms/feature	HPO phenotypic term IDs	Current case	Number of reported patients having the phenotype	Total cases: 29^b^ (%)
1	Sex		Male	Female: 17; male: 11	
2	Gestation weeks		41.5	Average: 38.7	
3	Birth weight		3.6	Average: 3.4	
4	Age		10	Average: 11.3	
5	Intellectual disability^a^	HP:0001249	+	19	20 (95)
6	Hypotonia	HP:0001252	+	22	23 (79)
7	Delayed ability to walk	HP:0031936		18	18 (62)
8	Global developmental delay (at various levels of severity)	HP:0001263	+	18	19 (66)
9	Seizure	HP:0001250		16	16 (55)
10	Absent speech	HP:0001344		13	13 (45)
11	Strabismus	HP:0000486		10	10 (35)
12	Delayed speech and language development	HP:0000750	+	9	10 (35)
13	Autistic behavior	HP:0000729		8	8 (28)
14	Impaired toileting ability	HP:0031061		8	8 (28)
15	Broad forehead	HP:0000337		6	6 (21)
16	Inability to walk	HP:0002540	+	6	7 (24)
17	Micrognathia	HP:0000347		5	5 (17)
18	Pain insensitivity	HP:0007021		4	4 (14)
19	Ataxia	HP:0001251		4	4 (14)
20	Delayed ability to sit	HP:0025336	+	3	4 (14)
21	Nystagmus	HP:0000639	+	3	4 (14)
22	Short philtrum	HP:0000322		4	4 (14)
23	Bilateral talipes equinovarus	HP:0001776	+	2	3 (10)
24	Caesarian section	HP:0011410	+	2	3 (10)
25	Reduced eye contact	HP:0000817		3	3 (10)
26	Dysmetria	HP:0001310		3	3 (10)
27	Scoliosis	HP:0002650		3	3 (10)
28	Large earlobe	HP:0009748		3	3 (10)
29	Prominent nasal tip	HP:0005274		3	3 (10)
30	Deeply set eye	HP:0000490		3	3 (10)

Amblyopia-HP:0000646, Failure to thrive-HP:0001508, Plagiocephaly-HP:0001357 and Torticollis-HP:0000473 were observed in our patient and one more patient in the previous literature; the following phenotypes were also observed in our patient: Abnormality of the curvature of the vertebral column-HP:0010674, Aspiration-HP:0002835, Cerebral palsy-HP:0100021, Cutaneous finger syndactyly-HP:0010554, Delayed ability to crawl-HP:0033128, Delayed ability to roll over-HP:0032989, Delayed ability to stand-HP:0025335, Delayed gross motor development-HP:0002194, Dysphagia-HP:0002015, Eczema-HP:0000964, Elbow flexion contracture-HP:0002987, Esotropia-HP:0000565, Flexion contracture of finger-HP:0012785, Gastrostomy tube feeding in infancy-HP:0011471, Hamstring contractures-HP:0003089, Hyperventilation-HP:0002883, Hypopnea-HP:0040213, Knee flexion contracture-HP:0006380, Language impairment-HP:0002463, Limited ankle dorsiflexion-HP:0033526, Limited neck range of motion-HP:0000466, Lower extremity akinesia-HP:0033411, Lower limb asymmetry-HP:0100559, Metatarsus adductus-HP:0001840, Myopic astigmatism-HP:0500041, Nuchal cord-HP:0012498, Optic nerve hypoplasia-HP:0000609, Periventricular leukomalacia-HP:0006970, Pes cavus-HP:0001761, Plantar flexion contractures-HP:0008112, Pneumonia-HP:0002090, Poor head control-HP:0002421, Sleep apnea-HP:0010535, Tetraplegia-HP:0002445, Visual impairment-HP:0000505.

^a^The phenotype intellectual disability was counted among patients who were 5 yr old or >5 yr old.

^b^Twenty-eight patients were already reported with variants in *TRPM3* gene and ours makes it 29.

#### Birth History, Growth, and Family History

The boy was delivered at 41 1/2 wk of gestation cesarean section because of failure to progress and nuchal cord. At the time of his initial presentation in our center at 9 d of age, his head circumference was 39 cm (92nd percentile), his weight was 3.57 kg (45th percentile), and his length was 49 cm (13th percentile). His head circumference was 49.5 cm (55.55 percentile) at age 30 mo. At that time his weight was 8.6 kg (<3rd percentile *z*-score/SD −4.56) and his length was 80 cm (<3rd percentile *z*-score/SD −3.20). His latest weight and length remained beneath the 3rd percentile with *z*-score/SD of −3.65 for weight and −3.93 for height. The family has a history of high blood pressure but no conditions relevant to the current case.

#### Systemic and Neurologic

Plagiocephaly and concomitant torticollis likely associated with decreased mobility were noted at 3 mo. By 5 mo of age, the boy was noted to have decreased interaction, poor visual tracking, and nystagmus. Brain magnetic resonance imaging (MRI) performed at 9 mo of age demonstrated periventricular leukomalacia. He was given the diagnosis of mixed (axial hypotonic/appendicular hypertonic) quadriplegic CP. He was hospitalized for aspiration pneumonia when he was 3 yr old. A gastrostomy tube (G-tube) was inserted. He was hospitalized again at the age of 4 yr for chronic otitis media associated with severe obstructive sleep apnea. His latest function was at Gross Motor Function Classification System (GMFCS) level V.

#### Musculoskeletal

At initial evaluation, the boy had bilateral talipes equinovarus and bilateral clasped thumbs. The thumbs were flexed at the metacarpophalangeal joints with decreased abduction; the thumb was clasped with webbing. The talipes equinovarus was treated with the Ponseti protocol of casting and percutaneous heel cord lengthening followed by external rotation bracing. Camptodactyly or flexion contracture of his long fingers was noted at an early age but has not required intervention. At his 2-yr visit, he was developing hamstring tightness and mild knee flexion contractures. With growth, he experienced recurrence of talipes equinovarus on the right side as well as the progression of his hamstring tightness and knee flexion contractures, which necessitated bilateral hamstring lengthening with distal femoral anterior hemiepiphysiodesis and right anterior tibialis tendon transfer with posterior tibialis lengthening at age 6. His knee flexion contractures improved. He developed hip adduction contractures. At age 9 yr, he underwent the removal of the hardware from his knees as well as the release of the abductor longus tendons about the hip. He has also developed elbow flexion contractures, which have not required surgical intervention.

### Genomic Analysis and Assessment of ACMG Criteria

**Figure 1.**
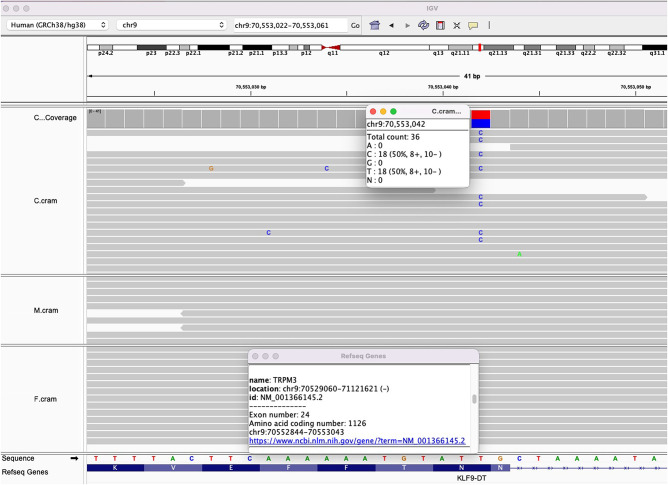
Trio-based whole-genome sequencing (WGS) revealed a de novo variant in the child, 9-70553042-T-C, that is, NM_001366145.2(TRPM3):c.3376A > G.

The DRAGEN Germline 3.9.5 pipeline (https://developer.illumina.com/dragen) aligned 1233 M reads to GRCh38 and reported a mean read depth of 34× with 90% of the bases with >20× coverage. The percentage of unmapped reads was 30% and the frequency of duplicated reads was 7.9%. The *TRPM3* variant NM_001366145.2:c.3376A > G, p.(Asn1126Asp), was identified in the proband and was not observed in either parent and thus is inferred to represent a de novo change. The variant was determined to be likely pathogenic based on the assessment of ACMG criteria (PS2, PM1, PM2, PP2, PP3, PP5) using InterVar (Li and Wang 2017). The variant is a de novo (ACMG class: PS2_supporting), located in the functionally important ion transport domain of the TRPM3 (PM1_moderate), absent in the control population cohort (PM2_supporting) (https://gnomad.broadinstitute.org/), multiple lines of computational evidence (PolyPhen-2: 0.999, MutationTaster: 1.000, and SIFT: 0.002) support it as a deleterious variant (PP3_supporting), and this variant was previously reported as a pathogenic in ClinVar (PP5_supporting). The read coverage for the variant was 36× with 50% of the bases containing the alternative base (Fig. 1). Because we did have genomic trios and used Integrated Genome Viewer (IGV) to exclude sample swap, in addition to kinship confirmation, Sanger confirmation was not performed and no other genetic or metabolic investigations were carried out. The variant was reported to ClinVar as SCV004021928.

**Figure 2.**
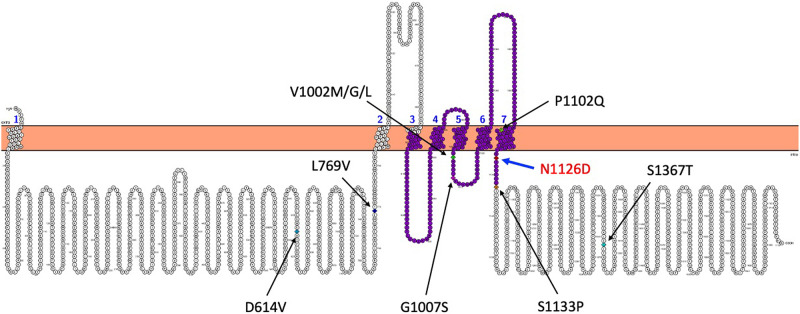
Illustration of human TRPM3 protein generated with PROTTER (Omasits et al. 2014) from the TRPM3 sequence (Q9HCF6-3, MANE-select sequence version). N1126D is the de novo variant identified in the current case and other previously known variants (listed in Table 2) have been mapped in the transmembrane domain and cytoplasmic domain of the TRPM3. The ion transport domain 891–1133 (residues from 904 to 1146 was on the TRPM3 canonical sequence, UniProt Q9HCF6) highlighted in purple contains seven of the 10 variants (or five of the eight variant positions) identified to date including the variant, N1126D, reported in this study.

**Figure 3.**
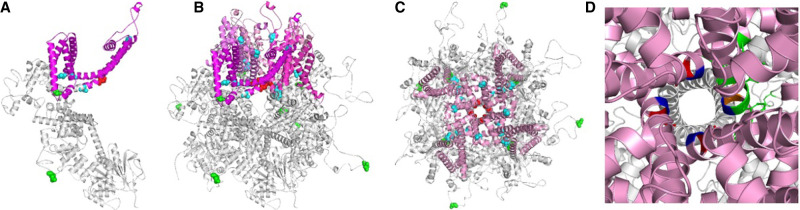
Three-dimensional structure and molecular interactions in human TRPM3 protein: (*A*) The TRPM3 protein represented by one chain; (*B*) homotetramer form of the TRPM3 channel, in a side view showing ion transport domain; (*C*) the TRPM3 protein from top view. In *A*–*C*, the variant position observed in the present study (N^1126^) is displayed in red; variant positions P^1102^, V^1002^, G^1007^, and S^1133^ that are present in ion transport domain are shown in cyan; variants from cytoplasmic domain (D^614^ and L^769^) are displayed in green color. (*D*) Corresponding view of the variant protein with Asp^1126^ shown in orange in one of the four chains along with interacting amino acids highlighted in green and neighboring Asn^1125^ in blue. The ion transport domain is displayed in magenta in *A* or four different shades of magenta to represent four chains of TRPM3 in *B* and pink in *C* and *D*.

The variant position was located in the ion transport domain of TRPM3 (Table 2; Figs. 2 and 3). Human TRPM3 is structurally a homotetrameric protein, and its ion channel structure has not been resolved. Therefore, we modeled its three-dimensional structure using Trpm3 of *Mus musculus* (PDB ID: 8DDT) as the template. The residue affected by the variant, Asn^1126^, is localized in the ion transport domain and more specifically present at one end of the S6 segment that forms the pore domain along with S5 segment (Figs. 2 and 3). The protein stability of wild-type and variant TRPM3 was assessed with DynaMut2 (Rodrigues et al. 2021), revealing a predicted protein stability change (ΔΔ*G*_stability_) of −0.25 kcal/mol, consistent with destabilization of TRPM3 structure by p.(Asn1126Asp).

**Table 2.** p.(Asn1126Asp) and previously reported *TRPM3* variants

Variant as reported in original publication	Mutant protein as reported in original publication	Variant in NP_001353074.1 (UniProt ID—Q9HCF6-3)	Count
NM_001366145.2:c.3376A > G	NP_001353074.1:p.(Asn1126Asp) (UniProt ID—Q9HCF6-3)	p.(Asn1126Asp)	Present case: 1; literature: 2
NM_020952.4:c.2509G > A	NP_066003.3:p.(Val837Met)	p.(Val1002Met)	16
NM_020952.4:c.2810C > A	NP_066003.3:p.(Pro937Gln)	p.(Pro1102Gln)	1
NM_020952:c.3605G > C	NP_066003.3:p.(Ser1202Thr)	p.(Ser1367Thr)	1
NM_001366145.2:c.1841A > T	NP_001353074.1:p.(Asp614Val)	p.(Asp614Val)	1
NM_001366145.2:c.2305C > G	NP_001353074.1:p.(Leu769Val)	p.(Leu769Val)	1
NM_001366145.2:c.3004G > T	NP_001353074.1:p.(Val1002Leu)	p.(Val1002Leu)	1
NM_001366145.2:c.3005T > G	NP_001353074.1:p.(Val1002Gly)	p.(Val1002Gly)	1
NM_001366145.2:c.3019G > A	NP_001353074.1: p.(Gly1007Ser)	p.(Gly1007Ser)	3
NM_001366145.2:c.3397T > C	NP_001353074.1: p.(Ser1133Pro)	p.(Ser1133Pro)	1

Transcripts and variants as reported in the original publications and the corresponding positions in NP_001353074.1, which was used as the protein isoform of reference for Figures 2 and 3.

## DISCUSSION

Cerebral palsy may be defined as a disorder of movement and posture that results from nonprogressive disturbances to the developing brain. The motor deficits of CP are often accompanied by disturbances of sensation, perception, cognition, communication, and behavior, by epilepsy, and by secondary musculoskeletal conditions (Rosenbaum et al. 2007). CP is not a single disease but a group of etiologically heterogeneous disorders whose pathophysiology relates to an injury to the developing brain in the prenatal through neonatal period (Patel et al. 2020). Recent genetic findings have shown that numerous Mendelian neurodevelopmental conditions may present with manifestations of CP, which have been termed “CP mimics” by some authors (Zouvelou et al. 2019). In this report, we described a child referred for treatment of CP in whom a de novo variant in the *TRPM3* was identified. To date, 10 *TRPM3* variants have been published in the medical literature in children with neurodevelopmental disorders, but our report is the first to describe CP manifestations in an individual with a pathogenic *TRPM3* variant.

Twenty-eight individuals have been previously reported to have ten different *TRPM3* variants (Table 2). Thus, together with the individual reported here, the clinical manifestations of 29 individuals with disease-associated *TRPM3* variants have been described. Human Phenotype Ontology terms observed in the present child and patients already reported in the literature with variants in *TRMP3* are provided in Table 1. Phenotypic abnormalities related to motor development were observed in 86% of patients (Delayed ability to walk: 18 and Inability to walk: 7), whereas language and speech impairment were observed in 79% of the patients (23 patients; Absent speech: 13 and Delayed speech and language development: 10). Further, Hypotonia (79%) and Global developmental delay (66%) were also frequently observed among patients with variants in *TRPM3*. Intellectual disability at various levels of severity (mild, moderate, or severe) was already reported in 95% of the patients (19 of 20 patients, whose age was 5 yr or more) with variants in *TRPM3*. In our patient, we observed severe intellectual delay. These observations together suggest that pathogenic variants in *TRPM3* are associated with intellectual disability. Among the previously reported variants, c.2509G > A resulting in p.(Val837Met) was reported in 16 out of the 29 individuals with phenotypic features including global developmental delay, intellectual disability, or seizures (Dyment et al. 2019; de Sainte Agathe et al. 2020; Gauthier et al. 2021; Kang et al. 2021; Lines et al. 2022).

The residue Val^837^ occupies a crucial position of *TRPM3* ion transport domain (S4–S5 linker region), a conserved helix that interacts with the TRP domain during gating (Dyment et al. 2019). Similarly, in the present case, the observed variant p.(Asn1126Asp) is located in the crucial ion transport domain, more specifically at the junction of TRP helix region and the S6 segment that forms the pore domain and main gate along with S5 segment of the *TRPM3* (Figs. 2, 3A–C). Using DynaMut2, we replaced Asn at 1126 with Asp in one of the four chains (chain A) of *TRPM3* protein structure and studied its impact on structural stability; this change appears to disturb the structure of protein in the pore-forming S6 domain (Fig. 3D). Residues Ala^1122^, Val^1123^, and Phe^1124^ from the pore-forming S6 region and residues Phe^1128^ and Phe^1129^ located in the TRP helix region are predicted to interact with Asp^1126^ in the variant p.(Asn1126Asp) TRPM3 protein. These results suggest that the variant p.(Asn1126Asp) may impact the original structure of the TRPM3 protein. Further, p.(Asn1126Asp) was previously reported to be a gain-of-function phenotype with increased basal activity leading to cellular calcium overload (Burglen et al. 2023). Hence, our report adds evidence for the association of the variant c.3376A > G in *TRPM3* with neurodevelopmental delay and is the first to document a variant in *TRPM3* in an individual with CP. Further data will be required to assess the strength of the causal relation between *TRPM3* variants and CP.

## CONCLUSION

We report a de novo missense variant in *TRPM3* in an individual with NEDFSS with manifestations of CP. Our report adds *TRPM3* to the list of variants in Mendelian disease–associated genes that have been observed in individuals with CP and confirms the pathogenicity of the variant p.(Asn1126Asp).

## METHODS

Written consent was obtained from both the parents of the child. The IRB under which this work was approved is WCG IRB.

### Whole-Genome Sequencing

The proband and his parents underwent WGS using the Illumina NovaSeq 6000 sequencing platform (Illumina). Libraries were constructed using the Illumina DNA PCR-Free Prep Tagmentation kit (Illumina) for paired-end parallel sequencing (2 × 150 bases).

### Variant Prioritization

We analyzed the WGS data using Exomiser, a bioinformatics software comprising a suite of algorithms for prioritizing disease-gene variants using random-walk analysis of protein interaction networks, clinical phenotype comparison with known patients based on HPO terms, and cross-species phenotype comparisons, as well as a wide range of other computational filters for variant frequency, predicted pathogenicity, and pedigree analysis (Smedley et al. 2015; Köhler et al. 2021). The variant c.3376A > G, p.(Asn1126Asp) was prioritized using Exomiser. We assessed the variant according to ACMG criteria (Richards et al. 2015). We report the variant according to the MANE-selected (Matched Annotation from NCBI and EMBL-EBI) *TRPM3* transcript NM_001366145.2 (NP_001353074.1) and mapped previously published variants (which had been reported according to NM_020952.4; NP_066003.3) to this transcript.

### Analysis of Protein Structure

We based our analysis on UniProt Q9HCF6-3, which corresponds to the MANE transcript. Three-dimensional structure of TRPM3 (Q9HCF6-3) was generated using SWISS-MODEL (Waterhouse et al. 2018) with *Trpm3* of *M. musculus* (PDB ID: 8DDT) as the template (Zhao and MacKinnon 2023); the mouse Trpm3 protein sequence has 93.2% of identity with that of human TRPM protein. Because 50% sequence identity between the target and the template protein is known to result in quality structural model (Vyas et al. 2012), the mouse Trpm3 protein with 93.2% sequence identity with human TRPM3 would serve as a reliable template. The model was generated as a homotetramer to visualize with better clarity the impact of the variant on the structure of the TRPM3. The effect of the missense variant p.(Asn1126Asp) on the protein stability was assessed using DynaMut2 (Rodrigues et al. 2021). The modeled structures of the wild-type and mutant *TRPM3* protein were visualized using PyMOL (The PyMOL Molecular Graphics System, Version 2.5.4).

## ADDITIONAL INFORMATION

### Data Deposition and Access

The variant c.3376A > G, p.(Asn1126Asp) was deposited to ClinVar (https://www.ncbi.nlm.nih./gov/clinvar/) under the accession number SCV004021928. We do not have permission to share raw data.

### Ethics Statement

Written consent and assent (when applicable) were obtained. The Institutional Review Board (IRB) under which this work is approved is the WCG IRB (WCG IRB Protocol #20212489).

### Acknowledgments

We thank the patient and his family for their contribution to our research.

### Author Contributions

P.N.R., A.M.B., and J.R.D. designed the study. A.G.G. performed next-generation sequencing. J.C.S., H.B., L.C., D.D., M.A.G., and P.N.R. performed bioinformatics analysis. A.M.B. and J.R.D. performed clinical analysis. A.C. and M.S. consented subjects and collected clinical data. E.M.R. performed clinical care. A.M.B. and M.S. performed scientific coordination. J.C.S. and P.N.R. drafted the manuscript, and all authors revised the manuscript.

### Funding

This study was supported by a grant from Shriners Hospitals for Children (Shriners-PR-21-03). Additional support was provided by the National Institutes of Health (NIH)/*Eunice Kennedy Shriver* National Institute of Child Health and Human Development (NICHD) (HD103805-02).

### Competing Interest Statement

The authors have declared no competing interest.

### Referees

Volkan Okur

Jean-Madeleine de Sainte Agathe
